# Enhanced Risk Aversion, But Not Loss Aversion, in Unmedicated Pathological Anxiety

**DOI:** 10.1016/j.biopsych.2016.12.010

**Published:** 2017-06-15

**Authors:** Caroline J. Charpentier, Jessica Aylward, Jonathan P. Roiser, Oliver J. Robinson

**Affiliations:** a Institute of Cognitive Neuroscience, University College London, London, United Kingdom; b Affective Brain Lab, Department of Experimental Psychology, University College London, London, United Kingdom

**Keywords:** Anxiety, Decision making, Emotion, Loss aversion, Memory, Risk aversion

## Abstract

**Background:**

Anxiety disorders are associated with disruptions in both emotional processing and decision making. As a result, anxious individuals often make decisions that favor harm avoidance. However, this bias could be driven by enhanced aversion to uncertainty about the decision outcome (e.g., risk) or aversion to negative outcomes (e.g., loss). Distinguishing between these possibilities may provide a better cognitive understanding of anxiety disorders and hence inform treatment strategies.

**Methods:**

To address this question, unmedicated individuals with pathological anxiety (*n =* 25) and matched healthy control subjects (*n =* 23) completed a gambling task featuring a decision between a gamble and a safe (certain) option on every trial. Choices on one type of gamble—involving weighing a potential win against a potential loss (mixed)—could be driven by both loss and risk aversion, whereas choices on the other type—featuring only wins (gain only)—were exclusively driven by risk aversion. By fitting a computational prospect theory model to participants’ choices, we were able to reliably estimate risk and loss aversion and their respective contribution to gambling decisions.

**Results:**

Relative to healthy control subjects, pathologically anxious participants exhibited enhanced risk aversion but equivalent levels of loss aversion.

**Conclusions:**

Individuals with pathological anxiety demonstrate clear avoidance biases in their decision making. These findings suggest that this may be driven by a reduced propensity to take risks rather than a stronger aversion to losses. This important clarification suggests that psychological interventions for anxiety should focus on reducing risk sensitivity rather than reducing sensitivity to negative outcomes per se.

Anxiety disorders constitute a major global health burden (1). They are characterized by disrupted emotional processing, working memory, and decision making (2, 3). Understanding impaired cognitive processing in anxiety disorders is important to identify targets for cognitive-based therapies for anxiety. Patients with anxiety frequently report difficulties concentrating and making decisions: demonstrating, for instance, increased risk avoidant behavior (4, 5, 6, 7) (see Table 1 for a summary of findings). Risk here is defined as uncertain situations in which the outcome probabilities are known, contrary to ambiguity, which involves unknown probabilities. Models of economic decisions, such as prospect theory (8, 9, 10), suggest that decision making under risk, in particular the commonly observed preference for sure outcomes over risky outcomes with equal or higher expected value, can be explained by a combination two phenomena: the diminishing sensitivity to outcome value as value increases, resulting in risk aversion, and the tendency to weigh potential losses more than potential gains, resulting in loss aversion. No study to date has, however, clearly distinguished risk from loss aversion in anxiety.

**Table 1:** Summary of Effects of Pathological Anxiety Disorders on Risky Decision Making

Study	Group	Task	Effect on Risk Taking: Patients vs. Controls
Maner *et al.*, 2007 (6), study 3	Anxiety disorders, mood disorders, learning/no Axis 1 disorders	RTBS (14-item version)	↓ in anxiety groups
			= in other groups
Mueller *et al.*, 2010 (7)	GAD	IGT (modified)	↓ (specific to decisions with small but consistent losses)
Giorgetta *et al.*, 2012 (5)	GAD, PAD	PGT (lotteries)	↓
Ernst *et al.*, 2014 (13)	GAD, SocPh, SAD (all adolescents)	Loss aversion	=
Galván and Peris, 2014 (14)	GAD, SocPh, SAD (children and adolescents)	Cups task (choice of safe vs. risky option)	↓ for losses
			= for gains
Butler and Mathews, 1983 (4)	GAD, MDD	Questionnaire	Overestimation of risk for negative events

Down arrow (↓) indicates decreased risk taking, and equals sign (=) indicates no effect.

GAD, generalized anxiety disorder; IGT, Iowa Gambling Task; MDD, major depressive disorder; PAD, panic attack disorder; PGT, probabilistic gambling task; RTBS, risk-taking behaviors scale; SAD, separation anxiety disorder; SocPh, social phobia.

Risk-taking behaviors in anxiety have been examined in a handful of studies. In one study (6), different groups of patients (anxiety disorder, mood disorder, learning disorder) and a group of healthy control subjects were administered a risk-taking questionnaire. Only anxious patients exhibited reduced levels of risk-taking relative to control subjects, suggesting that increased risk avoidance may be specific to anxiety. However, questionnaires are nonobjective and subject to well-established limitations including demand characteristics (11). In a modified version of the Iowa Gambling Task (7), patients with generalized anxiety disorder (GAD) exhibited increased avoidance of decks with accumulated low magnitude but consistent losses. However, the Iowa Gambling Task confounds multiple learning and decision-making processes and behavior could be explained by risk aversion, loss aversion, or learning. In another study (5), the authors addressed some of these concerns by administering a probabilistic gambling task that did not involve learning. Pathologically anxious individuals exhibited a strong reduction in their propensity to choose the riskier gambles relative to control subjects. However, once again, it cannot be determined from this design whether avoidance of these gambles is driven by enhanced aversion to risk, aversion to losses, or a combination. Finally, patients with anxiety tend to overestimate the risk of negative events (4), but it is unclear whether this might also extend to the positive domain. In sum, prior work assessing risk-taking behavior in anxiety is unclear.

There is also a strong hypothesis that loss aversion should increase with anxiety, given the associated negative biases in emotional and attentional processes, as well as the heightened sensitivity to large negative outcomes (2, 12). However, somewhat surprisingly, there are no published studies to date examining loss aversion in relation to anxiety in adult participants. One study looked at this question in adolescents (13) and found no difference in loss aversion between anxious and healthy adolescents. In other studies (5, 14) the gambling tasks used did not allow dissociating risk from loss aversion.

Here, we therefore adapted a previously published gambling task (15, 16) to clearly separate risk and loss aversion and explore performance in a group of healthy and unmedicated anxious individuals. By modeling participants’ behavior with a computational model derived from prospect theory, we were able to adequately estimate and separate these processes, hypothesizing that relative to healthy control subjects, pathologically anxious individuals would exhibit both increased risk and loss aversion.

## Methods and Materials

### Participants

Unmedicated individuals meeting criteria for GAD (*n =* 29) and matched healthy volunteers (*n =* 26) were recruited by advertisement. Data from 4 anxious and 3 control participants were excluded because of insensitivity to value in the gambling task (3 anxious, 1 control subject) or more than 10% of missed trials (1 anxious, 2 control subjects), making loss and risk aversion impossible to model. Final analyses included 25 pathologically anxious individuals (20 women, 5 men, mean age 25.2 ± 4.90 years [mean ± SD]) and 23 healthy control subjects (18 women, 5 men, mean age 25.74 ± 6.55 years; Table 2). Participants provided written informed consent and were paid for their participation. The study was approved by the University College London research ethics committee.

**Table 2:** Demographics, Questionnaire Scores, and Participants׳ Characteristics

	Pathologically Anxious Individuals (*n =* 25)	Healthy Controls (*n =* 23)	*t*_46_	*p*
Women:Men	20:5	18:5	—	—
Age, Years, Mean (SD)	25.20 (4.90)	25.74 (6.55)	–0.33	.75
Verbal IQ WTAR Score Out of 50, Mean (SD)	42.56 (4.42)	41.74 (5.75)	0.58	.57
STAI Trait Anxiety Score, Mean (SD)	55.24 (8.10)	30.00 (5.01)	12.85	<.001
BDI Score, Mean (SD)	16.96 (9.19)	1.57 (3.17)	7.62	<.001
Age of Onset of Anxiety, Mean (SD)	18.08 (5.99)	—	—	—
Number of Years With Anxiety, Mean (SD)	7.12 (5.85)	—	—	—
Current Major Depressive Episode, *n* (%)	13 (52)	—	—	—
Past Medication (Anxiolytic or Antidepressant), *n* (%)	2 (8)	—	—	—
Hospitalized for Anxiety or Depression, *n* (%)	1 (4)	—	—	—
Past Suicide Attempts, *n* (%)	1 (4)	—	—	—

Current diagnoses of other anxiety disorders within the anxious group (at the time of study) included: panic disorder (*n =* 5), panic attacks (not meeting criteria for panic disorder; *n =* 3), posttraumatic stress disorder (*n =* 3), agoraphobia (*n =* 2), obsessive-compulsive disorder (OCD; *n =* 1), compulsions and/or obsessions (not meeting criteria for OCD, *n =* 6), bulimia (*n =* 1), binge eating (not meeting criteria for bulimia; *n =* 2). Social anxiety and specific phobias were not assessed.

BDI, Beck Depression Inventory; STAI, State-Trait Anxiety Inventory; WTAR, Wechsler Test of Adult Reading.

### Procedure

Participants were prescreened by completing the trait section of the State-Trait Anxiety Inventory (17) online after expressing interest in participating in the study. High trait anxiety constitutes a vulnerability factor for anxiety disorders, with pathologically anxious individuals usually scoring above 50 (18, 19). A phone screening was conducted on participants scoring under 35 (prospective healthy control subjects) or above 50 (prospective anxious individuals) on the trait anxiety scale. Exclusion criteria included medication for psychiatric disorder (e.g., antidepressants) or consumption of cannabis in the last 30 days; consumption of any other recreational drug in the last week; alcohol or drug abuse in the last 6 months; current or past neurological disorder; current or past diagnosis of schizophrenia, bipolar disorder, attention-deficit/hyperactivity disorder, or learning disability. Any other current or past psychiatric diagnosis was also an exclusion criterion for the control group. All participants were fluent in English.

During their visit to the laboratory, all participants were first administered the Mini-International Neuropsychiatric Interview (20) to confirm eligibility. Because of high comorbidity with GAD (21, 22), major depressive disorder and other anxiety or anxiety-related disorders (panic, posttraumatic stress disorder, obsessive-compulsive disorder, agoraphobia, eating disorders) did not constitute exclusion criteria for the anxious group as long as criteria for current GAD were met. Participants also completed the Wechsler Test of Adult Reading (23) to measure verbal IQ.

Participants then practiced the emotional decision-making task by completing 1) a practice of the emotional memory task alone, 2) a practice of the gambling task alone, and 3) a practice on the combined task. The practice gambling task used a tailoring procedure (15, 16) to target each participant’s indifference point (IP; i.e., the difference in expected value between the gamble and the sure option such that the participant is indifferent between the two options). Specifically, it started with extreme trials where the values of the two options were clearly different; then these values were dynamically adjusted throughout the practice depending on the participant’s choices. The decisions were of two types: mixed gamble trials, for which the sure option was always £0 and the gamble involved a potential gain and a potential loss, and gain-only gambles, which involved a choice between a sure gain and a risky gamble with 50% chance of winning a higher amount and 50% chance of not winning anything (£0). Both risk and loss aversion can contribute to safe choice on mixed gambles, while only risk aversion contributes to safe choices on gain-only gambles. IPs for mixed gamble trials were as follows: for the anxious group, mean IP = 5.06 ± 2.30, median = 4.5, range = 2 to 10; for the control group, mean IP = 3.74 ± 2.37, median = 4, range = 0 to 9.5 (*t*_46_ = 1.96, *p =* .056). IPs for gain-only trials were as follows: for the anxious group, mean IP = 2.7 ± 3.53, median = 1.5, range = –2 to 8; for the control group, mean IP = 1.63 ± 3.17, median = 0.5, range = –2 to 8 (*t*_46_ = 1.10, *p =* .28).

The gambles were embedded in an emotional working memory task as part of a secondary aim of this study (Supplement), allowing us to investigate 1) whether gambling decisions are modulated by the emotional context as a function of anxiety [as suggested by Charpentier *et al.* (15)] and 2) whether working memory is modulated by the emotional context, and again whether this modulation varies with anxiety [as suggested by Charpentier *et al.* (16)].

**Figure 1:**
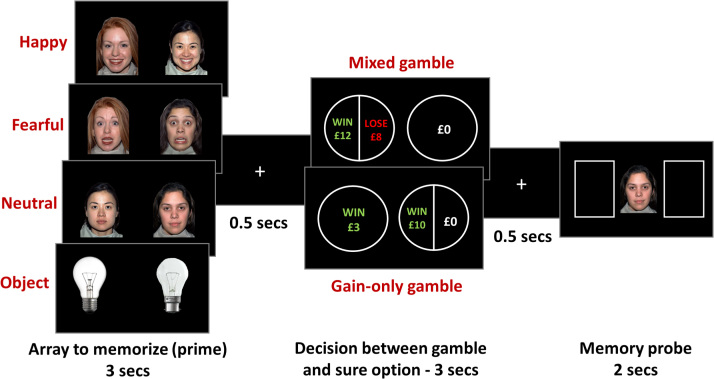
Trial design. On each trial, participants were first presented with a pair of faces (all happy, all fearful, or all neutral) or objects (light bulbs) and had 3 seconds to memorize it. They then had to decide whether to choose a sure option or a risky gamble. In mixed gamble trials, the sure option was always £0 and the mixed gamble involved a 50% chance to win the amount in green and a 50% chance to lose the amount in red. In gain-only gamble trials, the sure option was a small guaranteed gain, and the gamble involved a 50% chance to win a higher amount and a 50% chance to get £0. Finally, a probe from the first array was presented and participants had to report its position.

In each trial (Figure 1), participants were presented with a pair of stimuli belonging to one of the four conditions—fearful faces, happy faces, neutral faces, objects (light bulbs)—and were instructed to memorize their location. They then had to make a decision between a sure option and a risky 50-50 gamble. In each condition (happy, fearful, neutral, objects), there were 49 mixed gambles (7 × 7 matrix built) as well as 25 gain-only gambles (5 × 5 matrix), leading to a total of 296 trials, all randomly interleaved and split into four blocks. Both gamble matrices (Supplemental Figure S1) were centered on the participant’s IP estimated from the practice gambling task. Finally, one of the two stimuli from the initial pair appeared in the center of the screen and participants had to recall the initial left/right location of that stimulus.

After the task, participants completed the State-Trait Anxiety Inventory and the Beck Depression Inventory (24) (Table 2). As expected, both measures were significantly higher in anxious than control participants (trait anxiety [*t*_46_ = 7.62, *p <* .001], Beck Depression Inventory [*t*_46_ = 12.85, *p <* .001]).

### Behavioral Data Analysis

Behavioral data were analyzed in MATLAB (The MathWorks, Inc., Natick, MA), statistical tests performed in SPSS (version 22; IBM Corp., Armonk, NY), and Bayesian tests in JASP (version 0.7.1, JASP Stats, Amsterdam, the Netherlands) (25, 26). The propensity to choose the gamble was calculated separately for healthy control subjects and pathologically anxious individuals across all trials and compared using an independent two-sample *t* test. Second, it was calculated separately for mixed and gain-only gambles, and analyzed in a 2 × 2 analysis of variance with gamble type (within subjects) and group (between subjects) as factors. Reaction times, working memory accuracy, and missed trials were analyzed in a similar way (Table 3).

**Table 3:** Summary of Additional Task Variables

	Pathologically Anxious Individuals	Healthy Control Subjects	*t*_46_	*p*	Cohen’s *d*
RT_gamble_ (s)	1.294 (0.229)	1.319 (0.209)	–0.390	.699	0.113
RT_sure option_ (s)	1.134 (0.193)	1.250 (0.228)	–1.914	.062	0.553
Missed Gamble Responses (% Trials)	0.514 (0.787)	0.646 (1.125)	–0.477	.636	0.138
Working Memory Accuracy (Proportion Correct)	0.908 (0.11)	0.922 (0.047)	0.002 (arcsine)	.998	0.158
Missed Working Memory Responses (% Trials)	2.203 (2.717)	1.983 (2.183)	0.307	.760	0.089

For comparing working memory accuracy values (negatively skewed because of performance ceiling at 1) between groups, their values were arcsine transformed before running statistical tests. None of these other variables differed significantly between anxious and control groups, ruling out the possibility that they may have driven the observed difference in risk aversion.

RT, reaction time.

Given that each participant’s set of gamble values was centered on their IP from the practice gambling task, participants made decisions about differently valued gambles. Therefore, it was not possible to directly examine and compare gambling propensity as an index of risk taking. Instead, our computational modeling approach allowed us to adequately estimate risk and loss aversion for each participant, with the tailoring procedure being key to improving sensitivity of the model fitting procedure by ensuring that a maximum of decisions were close to each participant’s IP.

To estimate loss and risk aversion for each participant, a three-parameter prospect theory–derived model was used (8, 10, 27, 28). For each trial, the subjective utilities (*u*) of the gamble and the sure option were estimated using the following equations (with losses coded as negative values):

(Eq. 1) $$u\left(gamble\right)=0.5\times gain^{\rho }+0.5\times \lambda \times {\left(-loss\right)}^{\rho }$$

(Eq. 2) $$u\left(sure\right)=sure^{\rho }$$

λ represents loss aversion: λ > 1 indicates overweighing of losses relative to gains and λ < 1 the converse. ρ represents the curvature of the utility function, which reflects varying sensitivity to changes in values as value increases. If ρ < 1, the utility function is concave for gains and convex for losses, resulting in risk aversion (greater utility for a sure gain than for a risky 50-50 gamble with the same expected value); ρ > 1 indicates risk seeking.

These subjective utility values were then passed through a softmax function to estimate the probability of choosing the gamble on each trial (coded as 1 or 0 for choosing the gamble or the sure option, respectively), with the inverse temperature parameter µ:

(Eq. 3) $$P\left(gamble\right)=\frac{1}{1+e^{-µ[u\left(gamble\right)-u\left(sure\right)]}}$$

Best-fitting parameters (λ, ρ, and µ) were estimated using a maximum likelihood estimation procedure (Supplement). Three models were run using this procedure: λ, ρ, and µ estimated across all trials (three parameters; model 1), separately for each of the four emotion conditions (12 parameters; model 2), and only λ and ρ estimated for each emotion condition and µ estimated across all trials (nine parameters; model 3). The latter model was run because the 12-parameter model could not be reliably estimated (the MATLAB function solver exceeded the maximum evaluation limit of 400 attempts in 12 out of 48 subjects). Therefore the nine-parameter model was used to examine risk and loss aversion across the different emotion conditions (Supplemental Figure S3). Five comparison models were also estimated to ensure that our winning model performed better: constant, random, probability to choose the gamble on every trial (model 4), constant probability to choose the gamble on every trial equal to each participant’s average propensity to gamble on the whole task (model 5), only λ and µ estimated across all trials (model 6), only ρ and µ estimated across all trials (model 7), and only µ estimated across all trials (model 8). All models are presented in Table 4.

**Table 4:** BIC Scores and *R*^2^ Values Associated With the Different Prospect Theory Models and Comparison Models

Model Description	Number of Parameters	BIC	*R*^2^	Model Accuracy
Model 1: λ, ρ, and µ Estimated Across All Trials^a^	3	10,287^b^	.508	78.9%
Model 2: λ, ρ, and µ Estimated Separately for Each Emotion Condition	12	12,215	.543	79.9%
Model 3: λ and ρ Estimated Separately for Each Emotion Condition; µ Estimated Across All Trials	9	11,580	.534	79.5%
Model 4: Null Model^c^	0	19,583	0	50.0%
Model 5: Null Model^d^	1	16,869	.152	59.6%
Model 6: λ and µ (no ρ) Estimated Across All Trials	2	12,933	.367	70.8%
Model 7: ρ and µ (no λ) Estimated Across All Trials	2	16,839	.168	60.2%
Model 8: µ Only, Estimated Across All Trials	1	18,206	.084	55.1%

Model accuracy represents the percentage of choices correctly explained by the model, computed for each participant using their parameter estimates and averaged across participants. *R*^2^ and model accuracy values cannot be directly compared across models with different numbers of parameters.

a Main text model.

b Winning model (lowest Bayesian information criterion [BIC]).

c *p*_gamble_ = .5 on every trial.

d *p*_gamble_ = average propensity to gamble for that subject on every trial.

Model comparison was performed using Bayesian information criterion scores (29). Bayesian information criterion scores were summed across participants, with lower sum Bayesian information criterion scores indicating better model fit. Pseudo *R*^2^ were also calculated and averaged across participants, providing an estimate of the proportion of variance in the data explained by the model. Model accuracy was calculated as the proportion of choices correctly predicted by the model for each participant using their parameter estimates. Similarly, choice data were simulated using parameters from the winning model, separately for each group and each gamble type, to verify that participants’ propensity to gamble was accurately explained by the model. Finally, to test the reliability of our parameter estimates and ensure that varying IPs (and varying range of gamble values) across participants did not affect our findings, we ran simulation analyses to recover the parameters from simulated data (Supplemental Tables S1 and S2).

The distribution of both λ and ρ parameters was positively skewed (skewness values = 1.2 for λ and 1.7 for ρ), so they were log-transformed before running statistical tests. In addition, because risk aversion is highest for lowest values of ρ, –log(ρ) was taken as the final index of risk aversion. This allowed risk and loss aversion to be similarly distributed, with positive values of log(λ) and of –log(ρ) indicating loss aversion and risk aversion, respectively. Another approach to reduce the skewness in the distribution of parameter estimates is to use a maximum a posteriori estimation procedure (30). Running this procedure provided identical inference (Supplemental Table S3).

Risk and loss aversion estimates were compared between groups with independent two-sample *t* tests. In addition, Bayesian analyses (31, 32, 33, 34) were conducted to corroborate significant effects as well as to provide evidence for null effects (see Supplement). Additional exploratory analyses (Supplement) controlled for depression diagnosis (Supplemental Figure S2) and examining working memory performance across groups and its modulation by emotional cues (Supplemental Figure S4).

## Results

### Risk and Loss Aversion

Our prospect theory–derived model, estimating risk and loss aversion across all trials (model 1), rather than separately for each emotion condition (models 2 and 3), was the winning model, which also outperformed all other comparison models (models 4–8; Table 4). Estimated across all trials, the average loss aversion parameter (λ) across all participants was 2.039 ± 0.625, greater than 1, and consistent with loss-averse decisions and with previous literature suggesting that people weigh losses about twice as much as gains (10, 35, 36, 37). Risk aversion was also evident in people’s choices, with an average ρ parameter of 0.713 ± 0.458, lower than 1, and indicative of diminishing sensitivity to changes in value as value increases. Statistically, these were confirmed by one-sample *t* tests (against zero) on the log-transformed parameters, with loss aversion [log(λ)] and risk aversion [–log(ρ)] both significantly positive (loss aversion [*t*_47_ = 14.81, *p <* .001, Cohen’s *d* = 2.14] and risk aversion [*t*_47_ = 6.261, *p <* .001, Cohen’s *d* = 0.904]).

**Figure 2:**
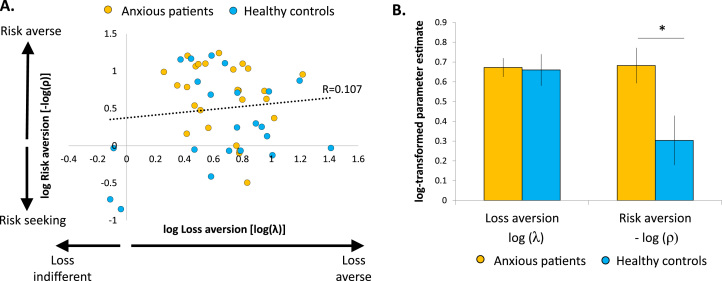
Risk and loss aversion parameter estimates. **(A)** Distribution of log-transformed parameter estimates. Positive values indicate risk aversion and loss aversion, respectively. **(B)** Mean estimates of loss and risk aversion, plotted separately for anxious and control groups. Error bars represent SEM. *p < .05 (two tailed).

The distribution of each parameter across individuals (Figure 2A) indicates that loss and risk aversion were not correlated across individuals (*r*_48_ = .107, *p =* .469), suggesting that distinct processes underlie risk and loss aversion and that the parameters are not trading off against each other in the model. To examine group differences in risk and loss aversion, both log-transformed parameters were analyzed separately and compared between groups (Figure 2B). Risk aversion was significantly higher in pathologically anxious individuals relative to control subjects (mean risk preference parameter ρ: anxious = 0.564 ± 0.313, control subjects = 0.875 ± 0.537; *t* test on log-transformed values [*t*_46_ = 2.491, *p =* .016, Cohen’s *d* = 0.720]), but there was no difference in loss aversion between groups (mean loss aversion parameter λ: anxious 2.013 ± 0.494, control subjects 2.067 ± 0.752; *t* test on log-transformed values [*t*_46_ = 0.141, *p =* .889, Cohen’s *d* = 0.041]). Critically, Bayesian analysis provided substantial evidence for a difference in risk aversion between groups (Bayes factor_10_ = 3.32) but favored the null over a group difference in loss aversion (Bayes factor_10_ = 0.29), enabling us to accept the null and say that there was no effect of group on loss aversion.

Examining the second-best performing model (model 3) to assess a possible role of emotional priming on decision making showed that risk and loss aversion were not affected by incidental emotional primes. There was also no difference between groups in how incidental emotions affected decision parameters (see Supplement and Supplemental Figure S3 for a complete analysis of model 3 parameters).

### Propensity to Gamble Is Accurately Explained by the Model

**Figure 3:**
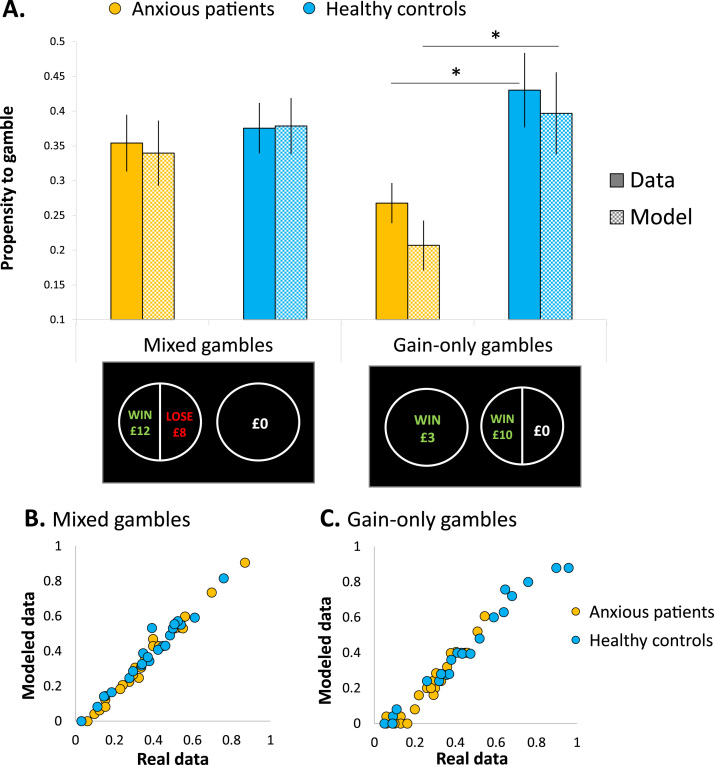
Propensity to gamble and model simulations. **(A)** The proportion of trials in which the gamble was chosen was calculated for each participant and each gamble type (mixed, gain only), then averaged separately for anxious and control groups (solid-filled bars). Model simulations were calculated in a similar way using each participant’s parameter estimates to calculate the utility difference between the gamble and the sure option on each trial, resulting in a simulated gamble choice if that estimated utility difference was positive and a simulated safe choice if it was negative. These simulated propensities to gamble were also calculated separately for each gamble type and averaged separately for anxious and control groups (grid-filled bars). Error bars represent SEM. *p < .05 (two-tailed *t* test). **(B, C)** Sensitivity plots depicting how well the modeled (or simulated) data correlated with the actual data, plotted separately for mixed gamble trials **(B)** and gain-only gamble trials **(C)**. Each data point represents an individual participant.

Across all participants, the average propensity to choose the gamble was 35.8% with no significant group difference (anxious individuals: 32.5 ± 16.1%, control subjects: 39.4 ±17.4% [*t*_46_ = –1.426, *p =* .161, Cohen’s *d =* 0.412]). However, when the type of gamble—mixed versus gain only—was added as a within-subjects factor, a significant gamble type by group interaction emerged (*F*_1,46_ = 5.196, *p =* .027, η_p_^2^ = .101; Figure 3A, solid-filled bars), such that propensity to gamble on mixed gamble trials did not differ between groups (*t*_46_ = –0.393, *p =* .696, Cohen’s *d =* 0.114), but anxious individuals gambled significantly less than control subjects on gain-only trials (*t*_46_ = –2.728, *p =* .009, Cohen’s *d =* 0.788). Note, however, that due to the tailoring process during the practice gambling task, the range of values used to build the gambles for the main task varied across participants; therefore, examining the proportion of chosen gambles may not reflect actual levels of risk-taking given that values may be different between subjects. These were instead reflected by risk and loss aversion parameters estimated from the prospect theory model (and shown in Figure 2B), taking into account the specific range of values for each participant. In turn, using these parameters to generate behavior on the task (i.e., a posterior predictive model) accurately explained participants’ propensity to gamble given their specific gamble set, as depicted by the grid-filled bars in Figure 3A. The gamble type by group interaction was replicated in the predicted data (*F*_1,46_ = 5.111, *p =* .029, η_p_^2^ = .100), with a significant group difference on gain-only gamble trials (*t*_46_ = –2.807, *p =* .007, Cohen’s *d =* 0.811) but not on mixed gamble trials (*t*_46_ = –0.626, *p =* .534, Cohen’s *d =* 0.181). In addition, sensitivity plots showing the modeled data plotted as a function of the actual data across individuals indicate a strong sensitivity of the model in capturing individual differences in the propensity to gamble data, both for mixed gambles (Figure 3B) and for gain-only gamble trials (Figure 3C).

## Discussion

This study demonstrated that relative to healthy individuals, pathologically anxious individuals exhibit enhanced risk aversion but similar levels of loss aversion. Originally, given the broad literature associating anxiety with more conservative decision-making styles (2, 3), we hypothesized that both risk and loss aversion would increase in pathological anxiety. Interestingly, however, only the first hypothesis was confirmed. Indeed, Bayesian analyses enabled us to accept the null hypothesis that pathological anxiety has no effect on loss aversion.

Anxious individuals show clear avoidance behaviors (6, 38, 39, 40, 41). The present data suggest that this behavior is driven by aversion to taking risks rather than aversion to losses per se. This is consistent with prior work demonstrating that pathologically anxious individuals show reduced tendency to take risks during gambling tasks (5, 7). Psychologically, a possible explanation for this increased risk-avoidance bias could stem from a bias in the evaluation of risk, with anxious individuals overestimating the risk of negative events (4). This would result in an overestimation of the probabilities of the so-called bad outcome of the gamble (regardless of whether that outcome is a smaller gain, a loss, or nothing, and therefore independent of loss aversion), leading to disengagement from risky decisions. An early model of anxiety suggested that intolerance to uncertainty is a pivotal feature of GAD (42). Decades of research on animal models of anxiety have also converged with human models, associating anxiety with altered responses to uncertainty, unpredictability, and/or uncontrollability of events and outcomes (43, 44). Intolerance to uncertainty likely plays a key role in the development and maintenance of pathological anxiety and may be an underlying mechanism of the increased aversion to risk observed in this study.

The hypothesis that loss aversion would also be enhanced in anxiety was rejected in the present study, as confirmed with Bayesian tests. The myriad of studies indicating negative attentional and emotional biases in anxiety (45, 46, 47, 48, 49, 50) led to the assumption that anxious individuals may give more weight to negative outcomes (in this case monetary losses) compared with healthy individuals. Yet, this had never been investigated by directly looking at loss aversion. Here, loss aversion was demonstrated in both healthy control subjects and pathologically anxious individuals: on average, participants weighed monetary losses approximately twice as much as monetary gains. However, this ratio was the same across both groups. This is consistent with a recent study in adolescents, which did not find any loss aversion difference between anxious and healthy adolescents (13). This finding is also in line with recent reports suggesting that induced anxiety in a sample of healthy participants, via threat of shock, did not influence high-level economic decisions, including loss aversion (16, 51). Although unexpected, this result may suggest that when the prospect of a loss or negative outcome is evaluated on its own, pathologically anxious individuals may be more sensitive than control subjects and report more negative judgments and affect; however, when they have to weigh this prospective loss against a prospective gain to make a decision, the degree by which they do so is similar to control subjects. Nevertheless, this is an important refinement of our understanding of the manifestation of pathological anxiety; it may be more about risk than loss.

With this study, we have addressed a significant omission in previous designs of risky decision-making task (5, 7, 14), where safer choices could be driven both by risk or loss aversion. Here we were able to reliably estimate both decision parameters within the same task and computational framework. However, a few limitations of the current study are worth mentioning. First, we note that the task was embedded in an emotional memory task and that decision making per se, without concomitant emotional priming and working memory could not be directly examined. Future studies should therefore aim to replicate the present findings using a simpler and more direct design. This would also permit the addition of loss-only trials to assess whether risk aversion in the gain and loss domains differ between groups. Second, despite our attempts at disentangling the effects of anxiety and depression (see Supplement for details), a possible effect of depression on risk/loss aversion remains possible and should be addressed in future studies explicitly designed to disentangle these effects. Similarly, higher sample sizes will be needed in future studies to separately assess the role of specific anxiety disorders such as panic, phobia, or social anxiety. Third, we note the sex unbalance in our sample, with far more female participants. Although this is generally expected in anxiety, a recent study has suggested that decision making may be impaired differentially in anxious men and women (52). Also, our anxious sample was recruited through advertisement in the general population, followed by telephone screening and structured face-to-face clinical interview, rather than through clinical services. Despite the advantage of being untreated, their behavior may differ from that of treatment-seeking patients encountered in clinical practice. Future work on treatment-seeking anxious patients may thus be useful to address possible differences. Finally, recent literature suggests that stress is an important modulator of decision making under risk (53, 54, 55, 56, 57), and may also interact with anxiety (58, 59). While we did not manipulate stress here, it would be interesting for future studies to investigate whether the effect of stress induction on risk aversion differ between pathologically anxious individuals and healthy control subjects.

Clinically, our results may be of importance given that pathologically anxious individuals frequently report difficulties making decisions in their everyday life, demonstrating, for instance, debilitating avoidance biases. In particular, these findings may help refine our understanding of successful cognitive behavioral therapy interventions like flooding (60, 61) and exposure therapy (62, 63) in which anxious individuals are encouraged to face their fears. Recent studies have indeed suggested that risk aversion is a relevant treatment outcome in anxiety (64) that should be directly targeted by cognitive behavioral therapy (65). The present findings indicate that the success of these interventions may not be so much about desensitizing individuals to the object of their fears (i.e., reducing loss aversion), but rather showing them that they can successfully navigate past them (i.e., they can take a risk and not come to harm). Cognitive behavioral therapy might therefore be about providing pathologically anxious individuals with a framework where they can take risks and still succeed (66) so that, ultimately, they reduce their overestimation of these risks.
