# Supplementary material for: Enhanced Risk Aversion, But Not Loss Aversion, in Unmedicated Pathological Anxiety
Source: Biol Psychiatry. 2017 Jun 15;81(12):1014–22. doi: 10.1016/j.biopsych.2016.12.010 (PMC5466268; doi:10.1016/j.biopsych.2016.12.010)
Supplement: Supplementary file 1 — Supplementary material [file mmc1.pdf]

Enhanced Risk Aversion, but Not Loss Aversion, in Unmedicated Pathological Anxiety

Supplemental Information

Supplemental Methods and Results

Payment procedure

Participants were paid in an incentive-compatible manner. They started the task with an endowment of £15. At the end of the task, 10 trials were randomly selected by the computer and the average outcome of these 10 trials was added or removed from the £15. The average payout was £18.14 ± 1.9 (range: [£14.50,£23.10]) and was not different between groups ($t(46)=0.35$, $P=0.73$).

Emotional face stimuli

Face stimuli were taken from the NimStim Face Stimulus Set (http://www.macbrain.org/resources.htm). All stimuli were resized to 200*300 pixels and displayed using Cogent 2000 (www.vislab.ucl.ac.uk/cogent.php) running under MATLAB.

Bayes Factor analyses

Contrary to classical frequentist approaches, Bayesian analyses can provide evidence in favor of the null model, by showing that the data are better explained by the null model than other models. Bayesian t-tests (1; 2) were used here to generate Bayes Factors (BF_{10}), which represent the evidence for a model of interest (model 1, which includes a main effect of subject and a difference between anxious and control groups in the variable tested) relative to a null model (model 0, which only includes a main effect of subject). We used the magnitude of the BF_{10} as an index to interpret the strength of evidence (3; 4): substantial evidence in favor of the model of interest when $BF_{10} > 3$; substantial evidence in favor of the null model when $BF_{10} < 0.33$.

Maximum likelihood estimation procedure

Our models capture loss and risk aversion effects by entering the difference between the utility of the gamble (main text Eq. 1) and the utility of the sure option (main text Eq. 2) in a softmax function that estimates the probability of choosing the gamble (main text Eq. 3). On mixed gamble trials, because both loss and risk aversion contribute to choice, both λ and ρ are present in the model, and the utility difference is as follows (the utility of the sure option is always 0):

$$u(gamble) – u(sure) = 0.5*gain^{\rho} + 0.5*\lambda*(-loss)^{\rho}$$

On gain-only trials, only risk aversion contributes to choice since there is not loss involved. Therefore, only ρ is present in the model, and the utility difference is as follows:

$$u(gamble) – u(sure) = 0.5*gain^{\rho} - sure^{\rho}$$

That utility difference is entered, for every trial, in the softmax function (main text Eq. 3). The resulting estimated probability of choosing the gamble is then compared to the participant's actual choice and converted into a negative loglikelihood value. The model-fitting procedure iterates in MATLAB (fmincon function) until it converges on the set of parameters that minimizes the negative loglikelihood (hence maximizing the likelihood of the data given that set of parameters).

Simulation analyses: Parameter estimates are reliable and independent of varying indifference points

Because of the staircase procedure used in the practice gambling task, each participant completed the main task with a range of gamble values tailored to their indifference point (IP). This means that participants were exposed to a different gamble set depending on their IP. To ensure our parameter estimates from the model were robust and did not depend on the gamble set used in the task, two types of simulation analyses were performed. First, we simulated choice data based on the parameter estimates of two typical participants (one from each group) who exhibited parameter estimates closest to the mean of their respective group. For each participant, two sets of gambles were constructed: one based on a low IP and one based on a high IP, with the rationale that parameter estimates should not vary with the gamble set used. Choices were then simulated 1000 times for each participant using their

parameter estimates, creating 1000 simulated datasets per IP and per participant; then parameters were estimated for each of these datasets using our standard modeling approach. The results of this analysis are presented in Table S1 and show that regardless of the IP used to construct the set of gambles, the parameters remain stable.

In the second analysis, we simulated choices on a single gamble set that encompassed all participants' indifference points. This resulted in a task set of 888 trials total (588 mixed gambles and 300 gain-only gambles) – instead of 296 trials for our regular task set centered on each participant's IP (196 mixed gambles and 100 gain-only gambles). Then we simulated choices on this extended task set 100 times for each of our 48 participants; and estimated model parameters for each simulated choice dataset. To ensure we could recover our main results, parameter estimates from the simulation were compared to parameter estimates from the actual data (Table S2). We find that across all participants, actual and recovered parameters were highly correlated ($r(48)>0.99$), and our main findings were replicated with the recovered parameters, namely higher risk aversion in anxious relative to control participants ($t(46)=2.496$, $P=0.016$) but no difference in loss aversion ($t(46)=0.118$, $P=0.91$). These analyses confirm the robustness of our computational modelling approach, which does not depend on the range of gamble values used in the task and allows the use of a tailoring procedure to increase sensitivity by ensuring a maximum of gambles are close to each participant's IP.

Maximum a posteriori (MAP) parameter estimates

The MAP procedure (5) estimates a first set of population parameters (averaged parameter estimates across all participants), which are then used as a prior for a second estimation. MAP parameter estimates are thus pulled towards the population mean, which helps reducing the impact of parameters with extreme values. Even though skewness was reduced (0.28 for λ and 1.4 for ρ), ρ values were still positively skewed, so we report statistical analyses on the log-transformed parameter in the main text. MAP parameter estimates are presented in Table S3, showing the same results as log-transformed parameters: enhanced risk aversion, but no difference in loss aversion, between anxious and healthy individuals.

Additional models

Two additional models were fit to the behavioral data. The first (Model 9) comes from the observation that the variance in the monetary amounts used in the mixed gambles is higher than the variance in the gain-only gamble amounts. Indeed, calculated across all amounts, variance in the mixed gambles was 110.9 ±50.2 (SD) while variance in the gain-only gambles was 41.61 ±39.8 (t-test on the difference: $P<0.001$). To take this difference in outcome scale into account, we estimated Model 9 that shared λ and ρ across all trials, but with μ estimated separately for mixed and gain-only trials. We find that μ values showed a trend towards being higher for mixed gamble trials ($\mu=7.01 \pm 8.80$) than for gain-only trials ($\mu=3.98 \pm 3.80$, paired-sample t-test on log-transformed parameter values: $t(47)=1.91$, $P=0.063$). However, this model did not perform substantially better than the main model (Model 1) described in the main text, despite the additional parameter (BIC score = 10,283 for Model 9 compared to 10,287 for Model 1). In addition, there was no change to the main findings using Model 9 (higher risk aversion in the anxious group relative to controls: $t(46)=2.48$, $P=0.017$; but no change in loss aversion: $t(46)=0.45$, $P=0.65$). We therefore retained the most parsimonious model (Model 1 - with only one μ parameter) as our main model.

The second model (Model 10) comes from the possibility that risk aversion could only be present in the gain-only trials, while only loss aversion would drive behavior on the mixed gamble trials. To test this possibility, we estimated Model 10 in which ρ is only defined on gain-only trials, while λ applies to losses in the mixed gamble trials, as usual. This resulted in both λ and ρ parameters being non-significantly higher in anxious individuals relative to controls (log-transformed loss aversion values = 1.39 ±0.68 for anxious group, 1.09 ±0.73 for controls, $t(46)=1.49$, $P=0.14$; log-transformed risk aversion values = 1.58 ±2.96 for anxious group, 0.32 ±0.76 for controls, $t(46)=1.98$, $P=0.054$), as well as a worse model fit (BIC = 10,294) than our main Model 1. This is most likely explained by the fact that mixed gamble decisions are best explained by a combination of risk and loss aversion, as in our winning Model 1. However, in Model 10, because ρ is not defined for mixed gambles, risk aversion is now captured by the loss aversion parameter.

Exploratory analyses controlling for depression scores

Anxiety is known to be highly comorbid with depression, with trait anxiety and BDI indeed highly correlated within the anxious group ($r(25)=0.617$, $P=0.001$, Figure S2A). Therefore,

we ran two exploratory analyses to examine whether the observed differences between anxious and control groups were indeed specific to anxiety or may be driven by depression.

For the first analysis, anxious individuals were divided into two groups according to whether they met criteria for current MDD in addition to GAD at the time of study. Indeed, while most individuals from the anxious group (24 out of 25) had experienced at least one depressive episode in the past, only about half (13 out of 25) met criteria for current MDD at the time of the study, thereby allowing us to examine the role of anxiety disorder in the presence or absence of depressive symptoms. A one-way ANOVA was then run on risk and loss aversion estimates, comparing the following three groups: anxious and depressed group (N=13), anxious non-depressed group (N=12), and controls (N=23). Means were then compared with post-hoc t-tests using the least significant difference (LSD) test. There was a significant effect of group on risk aversion (F(2,45)=3.853, P=0.029, η_p^2=0.146, Figure S2B) but no effect on loss aversion (F(2,45)=0.012, P=0.988, η_p^2=0.0005). For risk aversion, post-hoc LSD tests revealed increased risk aversion in the anxious non-depressed group relative to controls (mean difference = 0.509, P=0.009, Cohen's d=0.945), and no difference between the two anxious groups (mean difference = 0.252, P=0.24, Cohen's d=0.575). There was also no difference between the anxious and depressed group relative to controls (mean difference = 0.258, P=0.16, Cohen's d=0.460).

The second analysis used a continuous approach across the whole sample. Specifically, partial correlations were run with trait anxiety scores controlling for BDI scores and vice versa. We find that the correlation between risk aversion and trait anxiety remained significant while controlling for BDI (r(45)=0.328, P=0.024), while the correlation between risk aversion and BDI controlling for trait anxiety was not (r(45)=-0.194, P=0.19). For loss aversion, neither partial correlation was significant (correlation with trait anxiety controlling for BDI: r(45)=0.08, P=0.59; correlation with BDI controlling for trait anxiety: r(45)=-0.06, P=0.69). Standard bivariate correlation between variables (i.e. without controlling for a third variable) were also performed. Trait anxiety was significantly correlated with risk aversion (r(48)=0.31, P=0.032). None of the other correlations were significant (trait anxiety and loss aversion: r(48)=0.055, P=0.71; BDI and risk aversion: r(48)=0.16, P=0.29; BDI and loss aversion: r(48)=0.013, P=0.93).

These analyses suggest that if anything the increased risk aversion observed in anxious individuals relative to controls is driven by those anxious individuals who do not exhibit comorbid depressive disorder, arguing against an effect of depression in enhancing risk aversion. Even though this suggests that reduced risk-taking seems to be a specific feature of anxiety disorders, very little is known about economic decision-making biases in depression and this warrants further investigation.

Finally, in order to ensure that patients with comorbid MDD/GAD did not perform the task worse overall, we also examined the mu parameters between the same three groups (patients with GAD and MDD, patients with GAD only, and controls) we ran a similar one-way ANOVA as above but on the mu parameter values. This showed no group difference ($F(2,47)=1.44$, $P=0.25$), suggesting that patients with comorbid MDD/GAD did not perform worse than the other two groups overall (though we note that this subgroup comparison has relatively low power).

Emotional modulation of decision-making and working memory

A secondary aim of this study was to examine how emotional cues may alter economic decisions, as well as working memory performance, in pathological anxiety. To do so, gambling decisions in the task were embedded within an emotional working memory task, similar to previously published versions of the task (6; 7). Specifically, each decision to gamble or to choose the safe option was preceded by the short presentation of emotional (happy or fearful faces) or non-emotional (neutral faces or objects) cues, and followed by the retrieval of one of these cues (see Figure 1 in the main text). One of these previous studies has demonstrated that within a sample of healthy subjects, individuals with the lowest levels of dispositional anxiety exhibited an emotion-induced increase in loss aversion (6). Therefore, we hypothesized that individuals with pathological anxiety may show the opposite effect, modulating their decisions in response to emotionally arousing cues in a “non-optimal” direction, resulting in emotion-induced reduction in risk or loss aversion. In the second study (7), we showed that although anxiety induced by threat of shock in healthy participants did not alter gambling decisions, it improved memory for threat-congruent emotional cues in highly trait anxious individuals. Although the mechanisms by which threat of shock induce anxiety may be different from the processes underlying pathological anxiety,

we hypothesized that, relative to healthy controls, pathologically anxious individuals may exhibit improved recall of fearful faces and impaired recall of happy faces.

Impact of incidental emotions on decision-making

To investigate the role of emotional primes on gambling decisions, the propensity to choose the gamble over the sure option was calculated separately for each emotion condition and analyzed in a 4 (emotional prime: happy, fearful, neutral, object) by 2 (group: anxious, controls) ANOVA. Risk and loss aversion parameters were also analyzed in the same way after being estimated from our 9-parameter Prospect Theory model (Model 3 in main text Table 4). Indeed, this model performed better than the 12-parameter model (Model 2 in main text Table 4).

None of these decision-making related variables was influenced by the content of the prime (main effect of prime on propensity to gamble: $F(3,138)=1.459$, $P=0.228$, $\eta_p^2=0.031$, Figure S3A; on loss aversion: $F(3,138)=1.034$, $P=0.379$, $\eta_p^2=0.022$, Figure S3B; on risk aversion: $F(3,138)=0.877$, $P=0.455$, $\eta_p^2=0.019$, Figure S3C). The finding that Model 1, which estimates risk and loss aversion across all trials independent of emotion conditions, performs better than Models 2 and 3 (main text Table 4), is consistent with the null effect of emotion condition on risk and loss aversion. There was also no interaction between emotional prime and group on these variables (propensity to gamble: $F(3,138)=0.069$, $P=0.977$, $\eta_p^2=0.001$; loss aversion: $F(3,138)=1.653$, $P=0.180$, $\eta_p^2=0.035$; risk aversion: $F(3,138)=0.602$, $P=0.615$, $\eta_p^2=0.013$). Note that the increased risk aversion observed in anxious individuals relative to controls was present for each prime condition (Figure S3C).

This result is not consistent with our initial hypothesis based on (6) that anxious individuals may exhibit an emotion-induced reduction in loss or risk aversion. In addition, we did not replicate the finding that low anxious individuals show enhanced loss aversion in response to emotional cues. Several explanations could account for this difference between the two studies. First, the range of trait anxiety scores from the low anxious individuals in (6) – between 20 and 30 – was lower than the range of scores from the control group in the current study – between 22 and 39. It could therefore be that the emotion-induced increase in loss aversion observed in (6) is specific to individuals with extremely low levels of anxiety, consistent with our hypothesis of a link with resilience to anxiety. In addition, (6) was an

fMRI study, so there might be a sampling bias such that volunteers for brain scanning studies are generally less anxious. The conditions inside the fMRI scanner, such as noise, enclosed space and less opportunity for visual distraction may also have made the emotional manipulation procedure more potent in (6), possibly explaining the absence of effect in the current, behavioral study. Finally, a possibility could also be that the correlation with trait anxiety observed in (6), which was restricted to a healthy sample, does not extend to pathological anxiety, as cognitive processing may differ between high-trait anxious, but healthy, individuals, and pathologically anxious individuals.

Overall, the absence of effect of emotion or interaction between emotion and group on economic decisions is in line with the finding of (7), in which the same emotional cues, as well as anxiety induced by threat-of-shock, had no effect on gambling decisions and loss aversion. Other studies have also failed to show an effect of incidental emotions (in particular induced stress) on economic decisions (8; 9). While it is possible that in our particular design, the emotional faces were not potent enough to induce a strong emotion that would influence subsequent decisions, an alternative interpretation given these previous studies is that such high-level decision-making, and more generally deliberative, controlled processes, may be robust to incidental emotional influences. Future studies will be needed to distinguish between those two possibilities.

Impact of incidental emotions and anxiety on working memory

Memory performance was calculated for each participant as the propensity of correct recalls of the location of the stimuli presented in the center of the screen at the end of every trial (main text Figure 1), separately for each emotion condition. Working memory accuracy was 91.44% across all participants, and did not differ between groups (see main text Table 3). Because memory performance was close to ceiling and its distribution negatively skewed, recall accuracy values were arcsine-transformed before running statistical tests, as done previously (7).

A 4 (emotional prime: happy, fearful, neutral, object) by 2 (group: anxious, control) ANOVA on arcsine-transformed memory performance scores showed a significant interaction between emotion and group ($F(3,138)=2.995$, $P=0.033$, $\eta_p^2=0.061$; Figure S4A) as well as a significant main effect of emotion ($F(3,138)=4.184$, $P=0.007$, $\eta_p^2=0.083$). The main effect of emotion

was explained by greater recall of faces relative to objects across all participants (happy faces vs objects: t(47)=2.12, P=0.039, Cohen's d=0.306; fearful faces vs objects: t(47)=2.79, P=0.008, Cohen's d=0.403; neutral faces vs objects: t(47)=3.33, P=0.002, Cohen's d=0.481). The interaction, however, was driven by a decreased recall of emotional relative to neutral faces in anxious individuals (significant for fearful faces: mean decrease = -2.21% ±0.82, t(24)=-2.57, P=0.017, Cohen's d=0.514; non-significant for happy faces: mean decrease = -2.74% ±1.63, t(24)=-1.39, P=0.18, Cohen's d=0.278; paired t-tests run on arcsine-transformed data), but not in healthy controls, whose recall was not influenced by the emotional content of the faces (if anything it was increased: fearful vs neutral faces: +1.02% ±0.87, t(22)=1.53, P=0.14, Cohen's d=0.329; happy vs neutral faces: +0.03% ±0.73, t(22)=0.85, P=0.41, Cohen's d=0.177).

At first glance, this seems contradictory with the results of the previous study using threat of shock to alter memory performance (7), whereby threat-induced anxiety was found to improve recall of fearful faces but impair recall of neutral faces in high anxious subjects. To examine this apparent discrepancy closer, we have replotted the data from (7) in Figure S4, by performing a median split on trait anxiety scores within the sample of healthy controls used in that study, with high vs low anxious individuals mirroring the current anxious vs control groups. This analysis shows that during safe trials (Figure S4B), where no anxiety is induced as participants know that they are safe from shocks, the pattern of results is exactly the same as the finding of the current study (Figure S4A): reduced recall of fearful relative to neutral faces in high anxious individuals, but no difference in low anxious individuals. However, under threat (Figure S4C), this effect is normalized, such that threat (relative to safe) increases recall of fearful faces, but decreases recall of neutral faces in high anxious individuals.

The current finding on emotional modulation of memory recall in anxiety is therefore consistent with (7) and points towards a double dissociation of emotional influences on memory, such that emotion (in particular fear) impairs memory in pathologically anxious individuals, but tend to improve it in healthy controls. In addition, the findings of (7) suggest that threat may reduce this impairment in high anxious individuals.

We note, however, that the comparison between the current study and (7) should be taken with caution given that the high anxious individuals in (7) were not pathologically anxious,

and therefore had lower trait anxiety scores than the anxious group in the current study, and no history of depression.

In addition, a potential limitation of the present design (as well as the design in (7)) is that the recall task is relatively easy, with memory performance above 90% and close to ceiling. This possibly makes it harder to detect differences between emotional conditions. Future studies could investigate this question further by having more sensitive memory tasks.

Temporal effects on gambling behavior

In order to examine whether gambling behavior changed over the course of the task, and whether there was any group difference on this potential effect of time, we split the gambling data over 37 bins of 8 trials each. We then calculated, for each bin and each participant, the proportion of trials in which the gamble was chosen over these 8 trials. We ran a linear regression predicting propensity to gamble from time (coded from 1 to 37), and averaged the resulting regression coefficients (beta parameters) across participants. We found that there was a significant negative effect (mean beta=-0.0021 ±0.0046 (SD), t(47)=-3.14, P=0.001), such that the propensity to gamble decreased over the course of the task. However, this decrease was present in both groups (mean beta for anxious group=-0.0025 ±0.0052, t(24)= -2.46, P=0.021; mean beta for controls=-0.0016 ±0.0039, t(22)=-1.93, P=0.066) and there was no difference between groups (t(46)=0.74, P=0.46). This suggests a general tendency by which risk-taking in general decreases over the course of the task, regardless of anxiety.

Supplemental Tables

Table S1. Results of simulation analyses showing robust recovery of parameter estimates from more extreme set of choices on two example subjects

		IP mixed	IP gain-only	Mu	Lambda	Rho
**Example anxious participant**	Actual data	5	2.5	4.791	2.169	0.477
	Simulated data on **low** IP matrix (±SD from 1000 simulations)	2	0.5	4.989 (0.824)	2.175 (0.118)	0.478 (0.032)
	Simulated data on **high** IP matrix (±SD from 1000 simulations)	8	4.5	4.899 (0.660)	2.172 (0.074)	0.478 (0.017)
**Example control participant**	Actual data	4.5	1	2.725	2.145	0.783
	Simulated data on **low** IP matrix (±SD from 1000 simulations)	1.5	-1	2.841 (0.553)	2.153 (0.122)	0.785 (0.048)
	Simulated data on **high** IP matrix (±SD from 1000 simulations)	7.5	3	2.863 (0.632)	2.151 (0.090)	0.785 (0.035)

Table S2. Results of simulation analyses showing robust recovery of parameter estimates using the same set of choices (encompassing all indifference points) across all subjects

		Mu	Lambda	Rho
**Anxious group**	Actual parameter estimates (±SD from 25 subjects)	5.120 (4.124)	2.013 (0.494)	0.564 (0.313)
	Recovered parameter estimates from 100 simulations per subject (±SD from 25 subjects)	5.206 (4.238)	2.017 (0.499)	0.564 (0.315)
**Control group**	Actual parameter estimates (±SD from 23 subjects)	3.858 (4.502)	2.066 (0.752)	0.875 (0.537)
	Recovered parameter estimates from 100 simulations per subject (±SD from 23 subjects)	3.922 (4.562)	2.074 (0.757)	0.882 (0.549)

Table S3. Analysis of *maximum a posteriori* (MAP) parameter estimates

	Pathologically anxious individuals	Healthy controls	T(46)	P-value	BF_{10}
ρ (risk preference)	0.555 (0.296)	0.831 (0.459)	2.499	0.016*	3.37
λ (loss aversion)	1.988 (0.439)	2.025 (0.639)	-0.230	0.819	0.29

Supplemental Figures

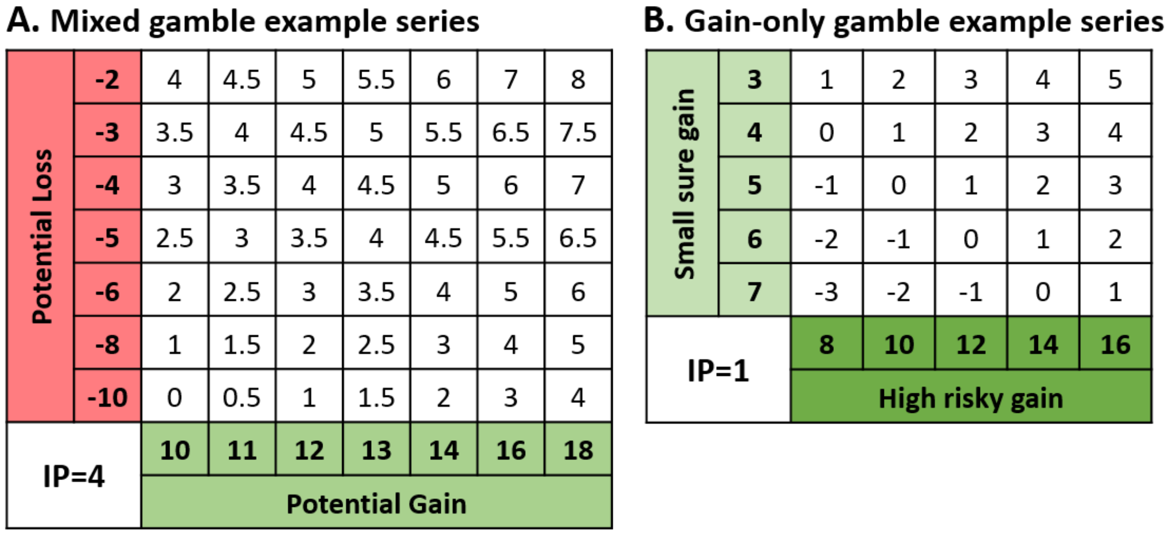

Figure S1. Example set of trials for a given indifference point. For mixed gamble trials (**A**), a matrix of 7*7 trials was used, each pairing a potential gain and a potential loss in a 50-50 gamble. The resulting gamble expected values presented in the table were calculated as 0.5*Gain + 0.5*Loss, here centered (matrix diagonal) on an indifference point of 4. For gain-only trials (**B**), a matrix of 5*5 trials was used, each pairing a small, sure gain and a high, risky gain. The values presented in the table are the difference between the gamble expected value and the sure option, calculated as 0.5*Risky Gain – Sure Gain, here centered on an indifference point of 1.

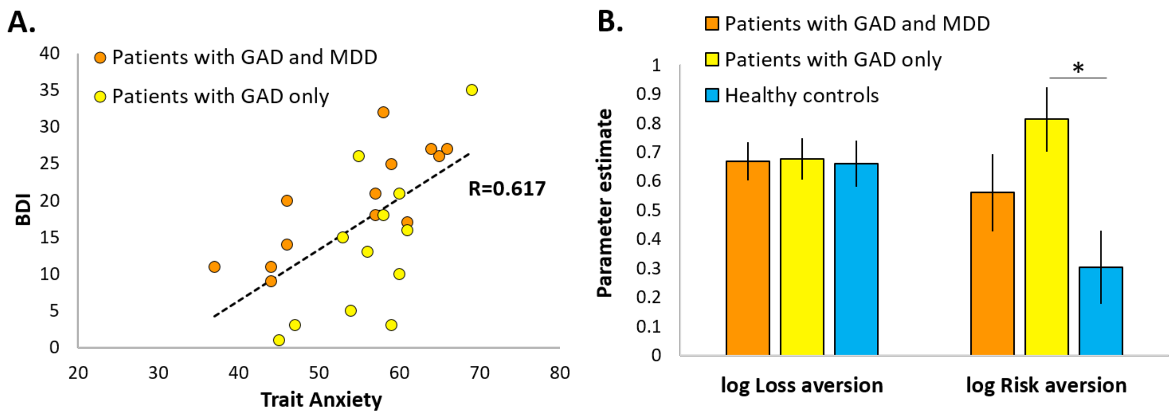

Figure S2. Controlling for depression levels in risk and loss aversion estimates. A. Despite their correlation with trait anxiety scores, variability in BDI scores and MDD diagnosis within the anxious group allowed examining differences in risk and loss aversion between anxious individuals who met criteria for current MDD and those who did not. **B.** These two subgroups did not differ in their level of loss and risk aversion; however, anxious individuals without MDD exhibited significantly higher risk aversion than controls, suggesting that increased risk aversion is driven by anxiety rather than depressive symptoms. Error bars represent SEM; post-hoc LSD tests: * P<0.05.

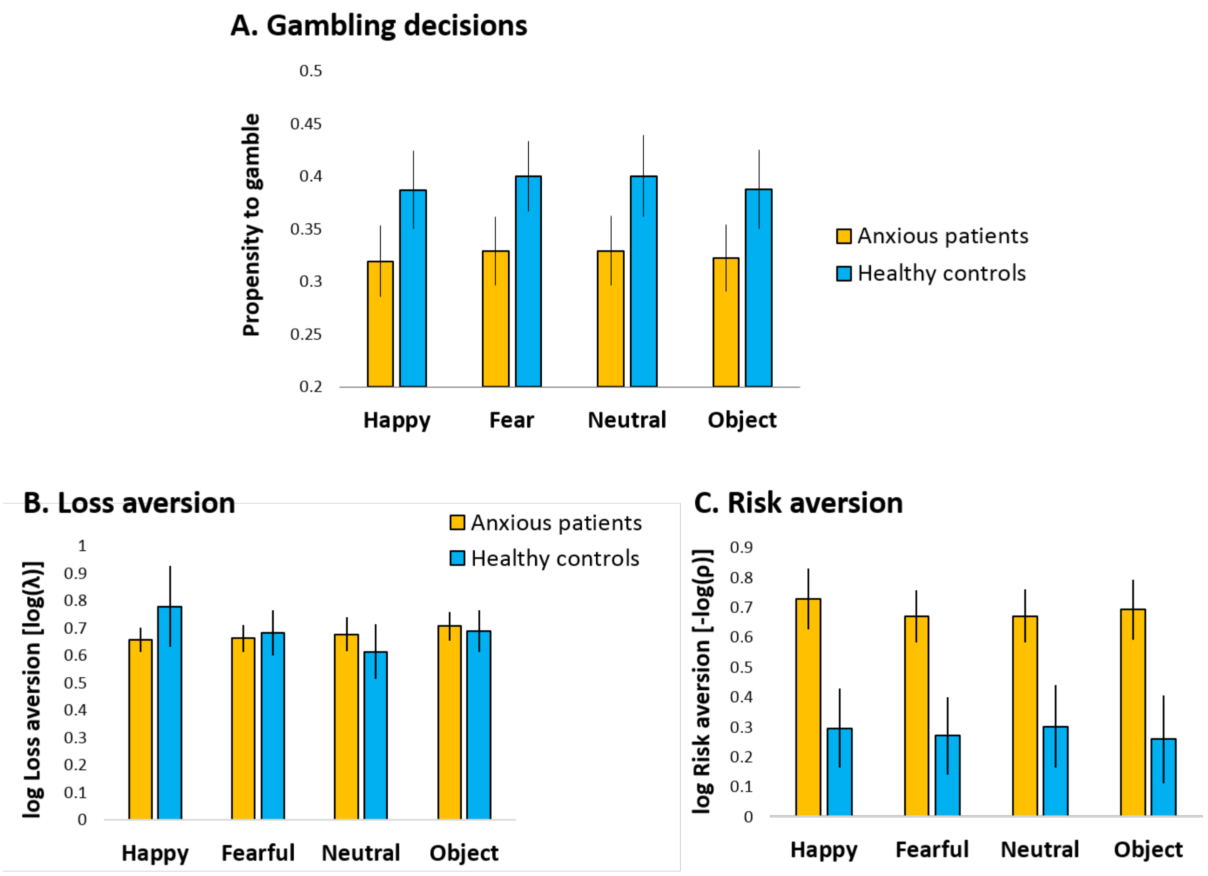

Figure S3. Impact of incidental emotional primes on economic decisions. The impact of emotional manipulation, through incidental presentation of visual prime prior to making a gambling decision, was examined on three variables: proportion of trials where the gamble is chosen (**A**), loss aversion (**B**) and risk aversion (**C**), calculated separately for each prime condition (happy faces, fearful faces, neutral faces, and objects) and averaged across anxious individuals and healthy controls. Error bars represent SEM; * P<0.05 two-tailed.

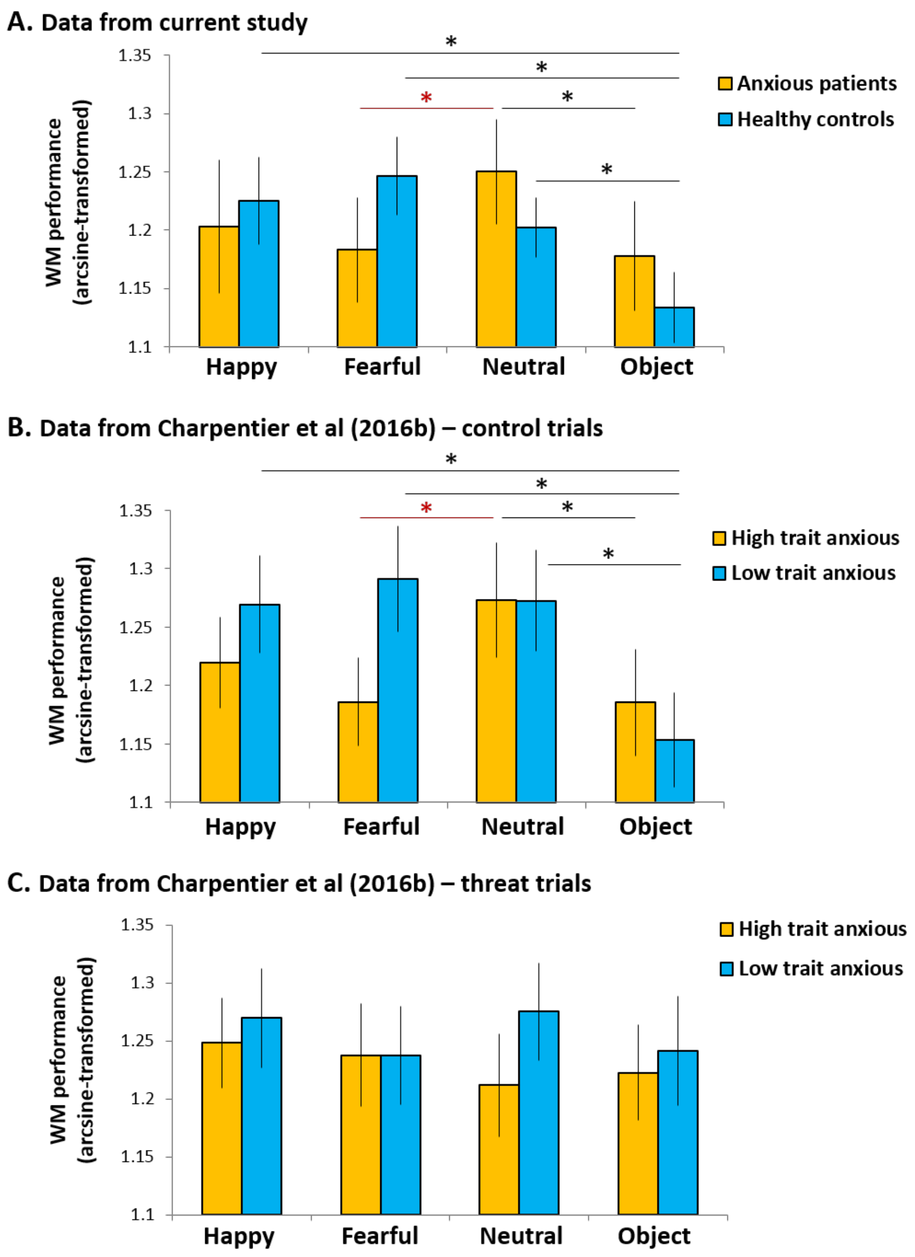

Figure S4. Impact of incidental emotional primes on working memory (WM) performance. Working memory accuracy scores were arcsine-transformed because negatively skewed then averaged separately for each prime condition. Data from the current study, plotted for anxious individuals and healthy controls (**A**), are compared with data from our recent study (7), in which healthy participants varying on trait anxiety scores performed the same emotional working memory task under threat of electric shock or safe from shock. Working memory performance data on control trials (safe from shock) is plotted in **B** separately for participants scoring high on trait anxiety (≥38) and participants scoring low (≤37), based on a median split on trait anxiety scores. The pattern of results in **A** and **B** are very similar. The effect of threat of shock reported in (7) and plotted in **C** was driven by a normalization of the differences observed in **B**, in particular improved memory performance for fearful faces and decreased performance for neutral faces in high anxious individuals. Error bars represent SEM; * P<0.05 two-tailed.

Supplemental References

1. Rouder JN, Speckman PL, Sun D, Morey RD, Iverson G (2009): Bayesian t tests for accepting and rejecting the null hypothesis. *Psychon Bull Rev*. 16: 225–237.
2. Rouder JN, Morey RD, Speckman PL, Province JM (2012): Default Bayes factors for ANOVA designs. *J Math Psychol*. 56: 356–374.
3. Jeffreys H (1961): *Theory of probability*, 3rd Ed. Oxford, UK: Oxford University Press.
4. Jarosz AF, Wiley J (2014): What are the odds? A practical guide to computing and reporting Bayes Factors. *J Probl Solving*. 7: 2–9.
5. Daw ND (2011): Trial-by-trial data analysis using computational models. In: Delgado MR, Phelps EA, Robbins TW, editors. *Decis Making, Affect Learn Atten Perform XXIII*. Oxford University Press, pp 3–38.
6. Charpentier CJ, De Martino B, Sim AL, Sharot T, Roiser JP (2016): Emotion-induced loss aversion and striatal-amygdala coupling in low-anxious individuals. *Soc Cogn Affect Neurosci*. 11: 569–579.
7. Charpentier CJ, Hindocha C, Roiser JP, Robinson OJ (2016): Anxiety promotes memory for mood-congruent faces but does not alter loss aversion. *Sci Rep*. 6: 24746.
8. Robinson OJ, Bond RL, Roiser JP (2015): The impact of threat of shock on the framing effect and temporal discounting: executive functions unperturbed by acute stress? *Front Psychol*. 6: 1315.
9. Engelmann XJB, Meyer F, Fehr E, Ruff XCC (2015): Anticipatory anxiety disrupts neural valuation during risky choice. *J Neurosci*. 35: 3085–3099.
